# “Je suis désolé, je parle français”: How English Hegemony Undermines Efforts to Shift Power in Global Health

**DOI:** 10.9745/GHSP-D-24-00201

**Published:** 2024-10-29

**Authors:** Shani Turke, Marieme Fall, Marie Ba, Sokhna Aminata Diop, Mohamed Ly, Elizabeth Larson, Elizabeth Arlotti-Parish, Sarah Nehrling

**Affiliations:** a Jhpiego, Baltimore, MD, USA.; b Independent consultant, Dakar, Senegal.; c Ouagadougou Partnership Coordination Unit, Dakar, Senegal.; d Engineering & Environment Services, Dakar, Senegal.; e EngenderHealth West and Central Africa, Abidjan, Cote d’Ivoire.; f Center on Gender Equity and Health, University of California San Diego, San Diego, CA, USA.; g Garabam Consulting, Livingston, TX, USA.

## Abstract

Le texte complet de l’article est aussi disponible en français.

## Abstract (precis)

English hegemony in the field of sexual and reproductive health works against efforts to shift power to local communities. To fully embrace locally led development, we must grapple with English language dominance and take actions toward becoming a more linguistically inclusive community.

## Key Messages

- While efforts to shift power in global health have been ongoing, professionals have done little to address English language hegemony in the field of sexual and reproductive health (SRH).
- English hegemony excludes populations and undermines localization efforts.
- English hegemony limits diverse representation in SRH, further burdens already disadvantaged groups, opposes priorities to localize and shift power, and stifles innovation.
- We propose immediate and long-term actions that organizations and individuals can take to move toward a more linguistically inclusive global SRH community.

## INTRODUCTION

At an international public health conference in 2022 that drew more than 3,000 sexual and reproductive health (SRH) professionals from around the world, a French-speaking researcher began his presentation by saying, “Je suis désolé, je parle français” (I’m sorry, I speak French). Despite the conference having heavily advertised English-French interpretation services, the interpretation was of poor quality, and at the start of his presentation, the researcher learned that interpretation was not working in his room. As the only Francophone presenter in a 4-person panel speaking to a largely Anglophone audience, many attendees left as he began his talk, presumably because they could not understand him. After the session, a Francophone attendee asked his colleague seated next to him to add English language instruction to his project’s budget, seeing limited English proficiency—and not poor interpretation services—as the true barrier to his full participation in the broader field.

As another example, an international organization based in the United States offers a competitive annual leadership course for its global staff in country offices around the world, noting English fluency as a requirement to apply. When staff raised concerns that the English requirement was exclusionary, a course organizer suggested non-Anglophones should instead view the requirement as motivation to improve their language skills via an English-only course.

These anecdotes provide salient examples of how English hegemony—defined as the domination of the English language over other languages—heightens inequality between Anglophone and Francophone SRH professionals in research and practice.^1^^,^^2^ Francophone nations comprise a significant percentage of countries that receive SRH donor funding, yet French speakers remain linguistically isolated.^3^^–^^6^

We, the authors of this commentary, comprise a diverse and multilingual group of Francophone African SRH professionals and their allies. As Francophones who regularly experience these inequalities, we argue that English hegemony limits the representation of diverse populations, further burdens already-disadvantaged groups, opposes global localization and power-shifting priorities, and stifles innovation by inhibiting the dissemination of learning published in languages other than English. We recognize that English hegemony is not unique to the SRH community and that Francophones are not the only language minority. However, we focus on French language exclusion for this commentary, given our positionality and the prioritization of Francophone countries in the global SRH agenda.^3^^–^^7^

We argue that English hegemony limits the representation of diverse populations, further burdens already-disadvantaged groups, and opposes global localization and power-shifting priorities.

Still, we recognize that in advocating for greater French language inclusion, we advocate for a colonial language that has also been used to exert global dominance and marginalize indigenous languages. Before World War I, French was the global lingua franca, not English.^8^ Furthermore, during the 150 years of French colonial rule in Africa, colonizers used French language instruction to advance French assimilationist policies.^9^ As African countries transitioned to independence in the mid-20th century, France promoted La Francophonie, an ideology of shared cultural identity among Francophone nations, to maintain control and combat English dominance.^9^^–^^11^ Given this history, we do not presume our positions reflect all opinions on English hegemony within the global SRH community.^12^ Instead, we advocate for improved French language inclusion as part of a greater discussion on the relationship between language and power within our profession. We look forward to dissenting opinions and the multilingual conversation to follow.

## ENGLISH HEGEMONY LIMITS REPRESENTATION OF DIVERSE POPULATIONS

Anglophone donors and international nongovernmental organizations (INGOs) that have historically implemented SRH programs perpetuate English dominance in the SRH field. FP2030 and KFF report that of the US$1.792 billion in international donor expenditures for family planning (FP) in fiscal year 2021, nearly 60% came from Anglophone governments and U.S.-based private donors. The U.S. Agency for International Development (USAID) and the Bill & Melinda Gates Foundation (BMGF), both of whom have long-prioritized SRH investments in Francophone Africa, contributed 47% of that funding.^3^^–^^7^^,^^13^^–^^15^ According to a 2024 review of the USAID FP and Reproductive Health Program, 14 of USAID’s 29 FP/reproductive health priority countries are French speaking, in addition to prioritizing the Francophone West Africa region as a whole.^7^ BMGF has also invested heavily in Francophone Africa, illustrated by their continued support of the Ouagadougou Partnership, which galvanizes momentum for FP in 9 Francophone African countries, and their FP investments in the Democratic Republic of the Congo.^4^^,^^6^^,^^15^

The dominance of the English language within the sector is juxtaposed with incredible language diversity among countries receiving SRH donor funding. In Africa, French is an official language in 48% of the continent’s 54 countries, compared to 37% and 13% of nations that have English and Portuguese as official languages, respectively.^16^ Beyond colonial languages, Africa is home to nearly one-third of the world’s total languages, and many people do not speak their country’s official language.^17^^,^^18^ For example, in the Central African Republic, only one-third of the population speaks French.^16^ In Niger, an estimated one-fifth of people older than 10 years are literate in French.^19^

Given this linguistic diversity, we should ensure that Africa’s official languages are, at a minimum, well represented in the global SRH community; however, English remains the lingua franca. While there are ways in which promoting a single language can facilitate global collaboration, it can also exclude non-Anglophones from the basic resources and systems needed to contribute equally. Furthermore, imposing English on those who may not even speak French as their country’s official language adds another barrier to participation for actual SRH program recipients, distancing us further from those we seek to support.

## ENGLISH HEGEMONY FURTHER BURDENS ALREADY DISADVANTAGED GROUPS

French colonial rule favored centralized control, direct rule, and assimilation.^20^^,^^21^ During African nations’ transitions to independence in the mid-20th century, France imposed exit terms designed to keep its former colonies financially and linguistically dependent, establishing a post-colonial Françafrique.^11^^,^^22^^,^^23^ Signed into effect in the late 1950s and early 1960s, these neocolonial agreements are still active in many countries. Some have suggested that this has contributed to Francophone Africa having weaker economies and government systems compared to Anglophone Africa.^20^ It is within this context that we note that Francophone Africa has some of the highest infant and maternal mortality rates in the world, high rates of child marriage and early childbearing, and a disproportionate burden of disease.^24^^,^^25^

In the global SRH community, we compound these disparities by imposing English throughout a project’s life cycle. Anglophone donors often only issue funding opportunities and review proposals in English. As donors expect evidence-based programming, we limit Francophones’ competitiveness by publishing evidence in English, even when the research focuses on Francophone Africa.^26^ Once projects are awarded, we increase Francophone professionals’ work volume and mental burden by requiring them to collaborate in English with Anglophone global staff.^2^ The authors of this commentary who lead teams in Francophone Africa have experienced staff feeling anxiety and stress when joining calls where they are required to speak English, fearing colleagues will view them as incompetent. Bilingual staff experience burnout and mental fatigue from the numerous requests to represent their team in English-only environments.

Regarding project learning, documentation, and dissemination, publications in French are disseminated less widely in an English-dominated field. This results in Francophone evaluators and researchers needing to choose between making findings broadly available by publishing in English or ensuring in-country stakeholders use them by publishing in French. English hegemony also leads to global conference organizers overlooking the translation and interpretation needs of language minorities, rendering entire sections of conference programs functionally inaccessible to non-Anglophones.^26^^,^^27^

## ENGLISH HEGEMONY OPPOSES GLOBAL PRIORITIES OF LOCALIZATION AND POWER-SHIFTING

In recent years, the SRH field joined larger development sector efforts to decentralize power and localize decision-making. While these are not new concepts, the COVID-19 pandemic and a surge in racial violence in the United States rejuvenated calls to shift power away from high-income nations toward donor-recipient countries.^28^^–^^31^ As part of the most recent effort, USAID overhauled its localization approach, including adding a strategy directing national and subnational partnerships in USAID-priority countries and increasing the target percentages of U.S. government foreign assistance going to local organizations.^32^ BMGF has also shifted its grantmaking structures to a more decentralized model, giving country and regional offices greater autonomy.^33^ Finally, INGOs have also pushed for more inclusive language and hiring practices to ensure their workforces better represent target populations.^30^^,^^34^ These efforts reflect principles of inclusivity, trust, and joint decision-making, which we applaud.

Paradoxically, it is within this context that we continue to exclude people from the localization movement because the discussion is in English. In the numerous articles published on decolonizing development in recent years, few have discussed the role of English hegemony, and those that have mentioned it do so only in the context of English domination over Indigenous languages.^30^^,^^34^ While it is important to acknowledge the detrimental role of British colonization in establishing English as a global lingua franca, it is not the only driver of English hegemony in the field. In describing it as such, we erase the experiences of Francophone SRH professionals who have their own complicated history with colonialism and who are often required to speak English for reasons unrelated to this history.

We continue to exclude people from the localization movement because the discussion is in English.

Language also influences international organizations’ decisions on where to establish regional offices. Institutions that have recently formed regional hubs in Africa without ensuring they are bilingual do little to increase decision-making power for Francophone countries. Rather, Francophones face the same language barriers only with new Anglophone counterparts operating in similar time zones. Such decisions ultimately reinforce a growing sentiment among Francophone SRH professionals that the localization movement is not for them.

## ENGLISH HEGEMONY STIFLES INNOVATION

There is a growing consensus that solving some of the SRH field’s most intractable problems requires complex, intersectional approaches.^28^^,^^29^^,^^35^^,^^36^ Even so, as a community, we continue to silo information by language and reinforce the fallacy that Anglophone knowledge systems are inherently superior.^29^^,^^37^ In doing so, we limit the talent pool for leadership positions and stifle cross-language learning that could lead to more sophisticated solutions.^35^^,^^38^

Research from the disciplines of linguistics and international relations documents a 70-year history of the United States intentionally promoting English as the language of international diplomacy to spread American values.^8^^,^^37^ Often referred to as linguistic imperialism, the global propagation of English after colonial rule in Africa has also devalued non-Anglo-American knowledge systems.^1^^,^^29^^,^^36^^,^^37^^,^^39^^,^^40^ As the 2 introductory anecdotes demonstrate, we see this perception of English-language superiority internalized even within our global SRH workforce.^2^ Professionals associate English with advancement and international prestige while reinforcing American values as the underpinnings of global development.^37^^,^^41^

Still, the field has lessons to learn from Francophone African history and scholarship on locally led development and power-shifting. Thomas Sankara, the revolutionary Burkinabè leader from 1983 to 1987, championed reinvesting in local goods and industry to develop Burkina Faso without foreign assistance.^42^ Sékou Touré has a complicated history as Guinea’s first president but also famously rejected Charles de Gaulle’s neocolonial terms of independence in 1958.^43^^,^^44^ Aminata Sow Fall and Mariama Bâ, 2 of the first published female Francophone African novelists, provide biting social commentary on Senegalese women’s status and the society’s propensity to reject indigenous values in favor of French culture.^45^^,^^46^ Finally, recent SRH commentaries from female Francophone professionals have made the case for how achieving gender equality is inextricably linked to greater Francophone inclusion.^47^^,^^48^ As we continue to grapple with operationalizing locally led development, including centering our work around the stated needs of women, it is problematic to ignore this seminal scholarship simply because it is published in French. In doing so, we miss opportunities for innovation and collective advancement.

## TOWARD A MULTILINGUAL CONVERSATION

As we transition to recommendations, we reiterate that we advocate for French language inclusion as a first step to broader language inclusivity. We do not have all the answers, so we present these recommendations to spark further discussion.

### What To Do Now

As a starting point for broader language inclusion initiatives in the field, we propose the following 4 key actions organizations and individuals can do now to support greater French-language inclusion.

#### 1. Use machine-assisted translation services for everyday electronic communications with non-Anglophone collaborators.

Multiple applications provide sufficiently accurate translations to support everyday electronic communications across languages. Some applications, such as DeepL, integrate into existing software and use machine learning to improve translations over time. If you do not speak the language of your collaborator, include the original text along with the translation in your communication. Although machine-assisted translation can be a functional alternative for everyday communications, we still recommend using professional human translators for official translations and external publications.

#### 2. Invest in bidirectional interpretation services by human interpreters.

Interpretation services have become more affordable, especially with the proliferation of virtual meeting platforms. Although interpretation services using artificial intelligence (AI) have significantly improved, they cannot accurately interpret the nuances and sensitivities of the language in the SRH field, particularly across cultures; thus, we do not currently recommend AI interpretation. Organizations should budget for bidirectional interpretation to ensure everyone can engage in their preferred language. Test interpretation and sound quality with interpretation firms before an event and retain the same interpreters for multiple engagements so they can learn discipline- and organization-specific language. This approach is particularly important in the SRH field, which has many sensitive and highly contextualized terms.

#### 3. Commit to incorporating document translation into routine project management processes.

As we previously discussed, the lack of systematic prioritization of document translation results in information gaps for Francophone populations. Even foundational SRH resources are often unavailable in French, or the translation is of poor quality. SRH professionals can address this by building key resources and final product translation into project implementation timelines. Anglophones also have much to learn from work implemented in Francophone contexts, so planning for translation from French to English is also critical.

#### 4. Design events as multilingual spaces.

Providing interpretation and translation services is a first step toward greater inclusion. To move beyond inclusion and toward belonging, organizations must design events and platforms to acknowledge language diversity and demonstrate to non-Anglophones that they are welcome. We can do this in a variety of ways, including the following.

To move beyond inclusion and toward belonging, organizations must design events and platforms to acknowledge language diversity and demonstrate to non-Anglophones that they are welcome.

- Translate slides and any other event materials and share them in advance with interpreters and participants.
- Establish and uphold norms that everyone should use interpretation services, not just non-English speakers. Anglophones in an English-dominated environment often do not expect spoken contributions in French and may miss what Francophones have to say if they are not listening to interpretation.
- Facilitate the event in multiple languages, alternating which language you use first or most frequently.
- Invite presenters and panelists who speak different languages and encourage them to present in the language in which they are most comfortable.
- Actively encourage exchange across languages through mixed language small groups and peer-to-peer learning.

### What We Need for Lasting Change

In addition to these more immediately accessible solutions, we offer 3 recommendations to donors and international organizations to promote broader structural change, some of which builds off previously published recommendations.^2^^,^^26^^,^^29^

#### 1. Promote inclusive systems of power via 4 principal mechanisms.

##### Listening to what Francophones want:

While this could take many forms, at its simplest, it involves listening sessions and surveys to hear directly from Francophones about how they want to be engaged and the role of language inclusion in these efforts. Investing in better interpretation and translation services could be a solution, as could paying for English language instruction for Francophones who want to learn English. Donors and international organizations should earmark funds for these solutions to implement them from the outset.

##### Making funding opportunities more accessible to non-English speakers and mandating high-quality language services throughout the project life cycle:

This means releasing requests for proposals in English and the country’s primary language(s) and allowing organizations to submit proposals in languages other than English. This also means making language considerations a part of grant requirements, including earmarking funds for bi/multilingual program design, ongoing linguistic support during implementation, and translation of project documentation.

##### Prioritizing hiring Francophone staff across all positions:

Cultural shifts within these organizations can drive more significant shifts in the field. Prioritizing hires with full working proficiency in French, or ideally, hiring more Francophones from donor-recipient countries, shifts the balance of power. Hiring directly from Francophone Africa can also indirectly address other equity issues, as underrepresented groups in the region are less likely to have opportunities to learn English compared to their peers. By building a multilingual workforce, we also build a workforce that better represents the communities where we work.

##### Ensuring that the SRH field’s major convening bodies and conferences are truly bilingual:

Simultaneous interpretation at these events is necessary but insufficient. Francophones and those who do not use English as a working language should be consulted and implicated in developing communications and event plans. Sessions should be offered in English and French, with presentations in both languages and bidirectional interpretation for question-and-answer sessions.

#### 2. Invest in systems that continuously encourage and assess the quality of translation and interpretation services.

There is often an assumption that offering translation and interpretation services is sufficient, resulting in confusion when Francophones do not meaningfully participate after service provision. As previously discussed, Anglo-American knowledge systems dominate the SRH field, propagating highly American cultural concepts like “autonomy” or “empowerment” that are not easily translated or conceptualized in French. As such, we argue that investment in language services requires continual and ongoing investment to ensure interpreters and translators are providing quality, nuanced translations of complex and evolving ideas. To do this well, organizations should invest in in-house or regularly contracted translators and interpreters, sufficiently prepare them for events, and manage a lexicon of SRH and rights-related and organization-specific terms, acronyms, and their standard translations.

#### 3. Institutionalize shifts toward a multilingual organizational culture by incorporating language issues into diversity, equity, inclusion, and belonging initiatives.

To undo over 50 years of English hegemony, we need sustained investment to build a culture where non-English speakers feel comfortable speaking their language and where Anglophones do not default to English as a lingua franca. With the increased focus on diversity, equity, inclusion, and belonging among donors and INGOs, we believe diversity, equity, inclusion, and belonging programs hold untapped potential to promote a shift toward valuing multilingualism rather than viewing it as an obstacle to overcome.

We believe diversity, equity, inclusion, and belonging programs hold untapped potential to promote a shift toward valuing multilingualism rather than viewing it as an obstacle to overcome.

## CONCLUSION

While using English as a lingua franca in the SRH field can facilitate greater cross-country exchange, this commentary articulates how English hegemony also contributes to excluding a significant proportion of SRH professionals who use French as their working language. This systemic exclusion further burdens already disadvantaged groups and ultimately runs counter to the localization movement to which so many of us are committed.

To combat English hegemony, we offer simple actions that individuals can take immediately, such as prioritizing and planning for translation and interpretation services and designing multilingual spaces to foster a sense of belonging. We also advocate for long-term structural changes, achievable by prioritizing Francophone representation in positions of power and ensuring global convenings are truly bilingual. In our ongoing efforts to improve SRH outcomes while addressing inequity within the SRH workforce, we should ensure that language inclusivity is also a part of the conversation.

## Supplementary Material

GHSP-D-24-00201_French_translation.pdf

GHSP-D-24-00201-Turke-Supplement.docx

## Notes

**Peer Reviewed**
