# Supplementary material for: “Je suis désolé, je parle français”: How English Hegemony Undermines Efforts to Shift Power in Global Health
Source: Glob Health Sci Pract. 2024 Oct 29;12(5):e2400201. doi: 10.9745/GHSP-D-24-00201 (PMC11521565; doi:10.9745/GHSP-D-24-00201)
Supplement: GHSP-D-24-00201-Turke-Supplement.docx [file GHSP-D-24-00201-Turke-Supplement.docx]

COMMENTAIRE

« Je suis désolé, je parle français » : comment l'hégémonie de l'anglais sape les efforts visant à transférer le pouvoir dans le domaine de la santé mondiale

**Titre abrégé :** L'hégémonie anglaise sape les efforts visant à transférer le pouvoir dans le domaine de la santé mondiale

Shani Turke,^[[1]](#footnote-1)^ Marieme Fall,^b^ Marie Ba,^c^ Sokhna Aminata Diop,^d^ Mohamed Ly,^e^ Elizabeth Larson,^f^ Elizabeth Arlotti-Parish,^a^ Sarah Nehrling^g^

**Message clé à retenir** : L'hégémonie de l'anglais dans le domaine de la santé sexuelle et reproductive va à l'encontre des efforts visant à transférer le pouvoir aux communautés locales. Afin d'adopter pleinement le concept du développement piloté par les acteurs locaux, nous devons nous confronter à la domination de la langue anglaise et prendre des mesures pour devenir une communauté plus inclusive sur le plan linguistique.

**Messages clés**

- Bien que des efforts aient été déployés pour transférer le pouvoir dans le domaine de la santé mondiale, les professionnels se sont peu attelés à remédier à l'hégémonie de la langue anglaise dans le domaine de la santé sexuelle et reproductive (SSR).
- L'hégémonie de l'anglais exclut les populations et sape les efforts de localisation.
- L'hégémonie de l’anglais limite la diversité de la représentation dans le domaine de la SSR, alourdit le fardeau des groupes déjà défavorisés, s'oppose aux priorités visant à localiser et à transférer le pouvoir, et freine l'innovation.
- Nous proposons des actions immédiates et à long terme que les organisations et les individus peuvent prendre pour progresser vers une communauté SSR mondiale plus inclusive linguistiquement.

Introduction

Lors d'une conférence internationale sur la santé publique qui s'est tenue en 2022 et qui a réuni plus de 3 000 professionnels de la santé sexuelle et reproductive (SSR) du monde entier, un chercheur francophone a commencé sa présentation en disant : « Je suis désolé, je parle français ». Bien que la conférence ait beaucoup promu les services d'interprétation anglais-français, l'interprétation était de mauvaise qualité, et au début de sa présentation, le chercheur a appris que l'interprétation ne fonctionnait pas dans sa salle. Etant le seul intervenant francophone d'un panel de quatre personnes s'adressant à un public majoritairement anglophone, de nombreux participant.e.s ont quitté la salle dès le début de son intervention, certainement parce qu'ils ne parvenaient pas à le comprendre. Après la session, un participant francophone a demandé à son collègue assis à coté de lui d'ajouter des cours d’anglais au budget de son projet, estimant que la non-maîtrise de l'anglais - et non la mauvaise qualité des services d'interprétation - était le véritable obstacle à sa pleine participation dans le domaine.

Dans un autre exemple, une organisation internationale basée aux États-Unis propose un cours de leadership annuel compétitif à son personnel international dans les bureaux pays répartis dans le monde entier, en précisant que la maîtrise de l'anglais est une condition d'admission. Lorsque le personnel a soulevé des préoccupations quant au caractère exclusif de l'exigence de l'anglais, un organisateur du cours a suggéré que les non-anglophones devraient plutôt voir cette exigence comme une motivation pour améliorer leurs compétences linguistiques à travers ce cours exclusivement offert en anglais.

Ces anecdotes illustrent de manière éloquente comment l'hégémonie de l'anglais - définie comme la domination de la langue anglaise sur d’autres langues - accentue les inégalités entre les professionnels anglophones et francophones de la SSR dans la recherche et la pratique.^1,2^ Malgré le fait que les pays francophones représentent un pourcentage important des pays qui reçoivent des fonds des bailleurs qui financent les programmes SSR, les francophones restent isolés sur le plan linguistique. ^3-6^

En tant qu’auteurs de ce commentaire, nous formons un groupe diversifié et multilingue de professionnels africains francophones de la SSR et de leurs alliés. En tant que francophones qui expérimentons régulièrement ces inégalités, nous soutenons que l'hégémonie de l'anglais limite la représentation des populations dans leur diversité, alourdit le fardeau des groupes déjà défavorisés, s'oppose aux priorités mondiales de localisation et de transfert de pouvoir, et freine l'innovation en empêchant la diffusion des connaissances publiées dans des langues autres que l'anglais. Nous sommes conscient.e.s que l'hégémonie de l'anglais n'est pas spécifique à la communauté des professionnel.le.s SSR, tout comme les francophones ne sont pas la seule minorité linguistique. Cependant, nous nous concentrons sur l'exclusion de la langue française dans ce commentaire, étant donné notre position et la priorité accordée aux pays francophones au niveau mondial dans le domaine de la SSR.^3-7^

Malgré cela, nous reconnaissons qu'en plaidant pour une plus grande inclusion de la langue française, nous défendons une langue coloniale qui a également été utilisée pour exercer une domination mondiale et marginaliser les langues autochtones. Avant la Première Guerre mondiale, le français était la langue universelle, et non l'anglais.^8^ En outre, pendant les 150 années de domination coloniale française en Afrique, les colonisateurs ont utilisé l'enseignement du français pour promouvoir les politiques assimilationnistes françaises.^9^ Alors que les pays africains accédaient à l'indépendance au milieu du XXe siècle, la France a promu la Francophonie*,* une idéologie d'identité culturelle partagée entre les nations francophones, afin de maintenir son influence et combattre la domination de l'anglais.^9-11^ Compte tenu de cette histoire, nous ne présumons pas que nos positions reflètent toutes les opinions sur l'hégémonie de l'anglais au sein de la communauté mondiale de la SSR.^12^ Au contraire, nous plaidons pour une meilleure inclusion de la langue française pour élargir la discussion sur la relation entre la langue et le pouvoir au sein de notre profession. Nous accueillons avec plaisir les opinions divergentes et la conversation multilingue qui en découlera.

L'hégémonie de l'anglais limite la représentation des populations DANS LEUR DIVERSITE

Les bailleurs anglophones et les organisations internationales non gouvernementales (ONGi) qui ont historiquement mis en œuvre des programmes de SSR perpétuent la domination anglaise dans le domaine de la SSR. FP2030 et KFF rapportent que sur les 1,792 milliards de dollars américains de dépenses des donateurs internationaux pour la planification familiale (PF) au cours de l'année fiscale 2021, près de 60 % provenaient de gouvernements anglophones et de bailleurs privés basés aux États-Unis. L’agence américaine pour le développement international (U.S. Agency for International Development, USAID) et la Fondation Bill & Melinda Gates (Bill and Melinda Gates Foundation, BMGF), qui ont depuis longtemps priorisé les investissements en SSR en Afrique francophone, ont contribué à 47 % de ce financement.^3-7,13-15^ Selon une revue du programme PF et de santé reproductive de l'USAID en 2024, 14 des 29 pays prioritaires pour la PF/ santé reproductive de l'USAID sont francophones, en plus de la priorité accordée à la région francophone de l'Afrique de l'Ouest dans son ensemble.^7^ La BMGF a également beaucoup investi en Afrique francophone, comme en témoignent son soutien continu au Partenariat de Ouagadougou, qui galvanise l’élan en faveur de la PF dans 9 pays d'Afrique francophone, ainsi que ses investissements dans la PF en République démocratique du Congo.^4,6,15^

La prédominance de l'anglais dans le secteur s’inscrit dans un contexte de grande diversité linguistique des pays qui reçoivent des fonds de donateurs SSR. En Afrique, le français est une langue officielle dans 48 % des 54 pays du continent, contre 37 % et 13 % des nations qui ont respectivement l'anglais et le portugais comme langues officielles.^16^ Au-delà des langues coloniales, l'Afrique abrite près d'un tiers de toutes les langues du monde, et de nombreuses personnes ne parlent pas la langue officielle de leur pays.^17,18^ Par exemple, en République centrafricaine, seul un tiers de la population parle le français.^16^ Au Niger, on estime qu'un cinquième des personnes âgées de plus de 10 ans sait lire et écrire en français. ^19^

Compte tenu de cette diversité linguistique, nous devrions veiller à ce que les langues officielles de l'Afrique soient, au minimum, bien représentées au sein de la communauté mondiale de la SSR ; toutefois, l'anglais reste la *lingua franca*. Bien que la promotion d'une langue unique puisse faciliter la collaboration mondiale, elle peut également exclure les non-anglophones des ressources et des systèmes de base nécessaires pour apporter une contribution égale. En outre, imposer l'anglais à des personnes qui ne parlent même pas le français comme langue officielle de leur pays ajoute un obstacle supplémentaire à la participation des destinataires des programmes SSR, ce qui nous éloigne encore plus de ceux et celles que nous cherchons à soutenir.

L'hégémonie de l'anglais alourdit le fardeau des groupes déjà défavorisés

Le régime colonial français a favorisé le contrôle centralisé, la gouvernance directe et l'assimilation.^20,21^ Lors de la transition des nations africaines vers l'indépendance au milieu du XXe siècle, la France a imposé des conditions de sortie conçues pour maintenir ses anciennes colonies dans une dépendance financière et linguistique, établissant ainsi une Françafrique postcoloniale.^11,22,23^ Entrés en vigueur à la fin des années 1950 et au début des années 1960, ces accords néocoloniaux sont encore en place dans de nombreux pays. Certain.e.s ont suggéré que cela a contribué à affaiblir les économies et les systèmes gouvernementaux de l'Afrique francophone par rapport à ceux de l'Afrique anglophone.^20^ Il est dans ce contexte que nous constatons que l'Afrique francophone présente certains des taux de mortalité infantile et maternelle les plus élevés au monde, des taux élevés de mariages d'enfants et de grossesses précoces, et une charge de morbidité disproportionnée. ^24,25^

Au sein de la communauté mondiale de la SSR, nous aggravons ces disparités en imposant l'anglais tout au long du cycle de vie d'un projet. Les bailleurs de fonds anglophones ne publient souvent des offres de financement et n'examinent les propositions qu'en anglais. Comme les donateurs attendent des programmes fondés sur des évidences, nous limitons la compétitivité des francophones en publiant ces évidences en anglais, même lorsque la recherche se déroule en Afrique francophone.^26^ Une fois les projets attribués, nous augmentons le volume de travail et la charge mentale des professionnels francophones en leur demandant de collaborer en anglais avec le personnel international anglophone.^2^ Les auteurs de ce commentaire qui dirigent des équipes en Afrique francophone, ont constaté que leur personnel ressentait de l'anxiété et du stress lorsqu'il se joignait à des appels où il devait parler anglais, craignant que les collègues ne le considèrent comme incompétents. Le personnel bilingue est victime d'épuisement et de fatigue mentale en raison des nombreuses sollicitations de représentation de leur équipe dans des environnements exclusivement anglophones.

En ce qui concerne l'apprentissage, la documentation et la diffusion des résultats des projets, les publications en français sont largement moins diffusées dans un domaine dominé par l'anglais. Les évaluateurs et les chercheurs francophones doivent en outre choisir entre publier leurs résultats en anglais pour les rendre accessibles au plus grand nombre et publier en français pour s'assurer que les parties prenantes dans le pays les utiliseront. L'hégémonie de l'anglais conduit également les organisateurs de conférences internationales à négliger les besoins des minorités linguistiques en matière de traduction et d'interprétation, rendant ainsi des sections entières des programmes de conférences fonctionnellement inaccessibles aux non-anglophones. ^26,27^

L'hégémonie de l'anglais s'oppose aux priorités mondiales de localisation et DE TRANSFERT DE pouvoir

Ces dernières années, le domaine de la SSR s’est associé aux efforts déployés par le secteur du développement pour décentraliser le pouvoir et localiser la prise de décision. Bien que ces concepts ne soient pas nouveaux, la pandémie de la COVID-19 et la montée de la violence raciale aux États-Unis ont ravivé les appels à transférer de pouvoir des pays à hauts revenus vers les pays bénéficiaires.^28-31^ Dans le cadre des efforts les plus récents, l'USAID a révisé son approche de la localisation, notamment en ajoutant une stratégie visant à encourager les partenariats nationaux et sous nationaux dans les pays prioritaires de l'USAID et en augmentant les pourcentages cibles de l'aide étrangère du gouvernement américain destinés aux organisations locales.^32^ La BMGF a également modifié ses structures d'octroi de subventions pour adopter un modèle plus décentralisé, en accordant une plus grande autonomie aux bureaux nationaux et régionaux.^33^ Enfin, les organisations internationales non gouvernementales (ONGi) ont également fait pression en faveur d'un langage et de pratiques d'embauche plus inclusifs afin de s'assurer que leur personnel représente mieux les populations cibles.^30,34^ Ces efforts reflètent les principes d'inclusion, de confiance et de prise de décision conjointe, que nous saluons.

Paradoxalement, c'est dans ce contexte que nous continuons d’exclure des personnes du mouvement de localisation parce que la discussion se déroule en anglais. Parmi les nombreux articles publiés ces dernières années sur la décolonisation du développement, peu ont abordé le rôle de l'hégémonie de l’anglais, et ceux qui l'ont mentionné ne l'ont fait que dans le contexte de la domination de l'anglais sur les langues autochtones.^30,34^ Bien qu’il soit important de reconnaître le rôle néfaste de la colonisation britannique dans l'établissement de l'anglais comme *lingua franca* mondiale, ce n'est pas le seul moteur de l'hégémonie de l'anglais dans ce domaine. En le décrivant ainsi, nous effaçons les expériences des professionnels francophones de la SSR qui ont leur propre histoire complexe avec le colonialisme et qui sont tenus de parler anglais pour des raisons sans lien avec cette histoire.

La langue influence également les décisions des organisations internationales quant à l'implantation de leurs bureaux régionaux. Les institutions qui ont récemment créé des centres régionaux en Afrique sans s'assurer de leur bilinguisme ne contribuent guère à accroître le pouvoir de décision des pays francophones. En revanche, les francophones sont confrontés aux mêmes barrières linguistiques, mais avec de nouveaux homologues anglophones opérant dans des fuseaux horaires similaires. De telles décisions renforcent le sentiment croissant des professionnels SSR francophones que le mouvement de localisation ne les concerne pas.

L'hégémonie de l’anglais FREINE l'innovation

Il existe un consensus croissant sur le fait que la résolution de certains des problèmes les plus difficiles à résoudre dans le domaine de la SSR nécessite des approches complexes et intersectorielles.^28,29,35,36^ Et pourtant, notre communauté continue à cloisonner l'information en fonction de la langue et à renforcer l'idée fausse que les systèmes de connaissance anglophones sont intrinsèquement supérieurs.^29,37^ Ce faisant, nous limitons le vivier de talents pour les postes de direction et nous freinons l'apprentissage inter linguistique qui pourrait conduire à des solutions plus sophistiquées. ^35,38^

Des recherches menées dans les domaines de la linguistique et des relations internationales montrent que, depuis 70 ans, les États-Unis ont entrepris de promouvoir l'anglais comme langue de la diplomatie internationale afin de propager les valeurs américaines.^8,37^ Souvent qualifiée d'impérialisme linguistique, la propagation mondiale de l'anglais après la colonisation de l'Afrique a également dévalorisé les systèmes de connaissance non anglo-américains.^1,29,36,37,39,40^ Comme le montrent les deux anecdotes présentées dans l'introduction, cette perception de la supériorité de la langue anglaise est internalisée même au sein de notre personnel mondial de de la SSR.^2^ Les professionnels associent l'anglais à l'avancement et au prestige international, tout en renforçant les valeurs américaines comme la base du développement mondial.^37,41^

Pourtant, le domaine a des leçons à tirer de l'histoire et es recherches de l'Afrique francophone sur le développement piloté par les acteurs locaux et le transfert du pouvoir. Thomas Sankara, dirigeant révolutionnaire burkinabé de 1983 à 1987, prônait le réinvestissement dans les biens et industries locaux pour développer le Burkina Faso sans aide étrangère.^42^ Sékou Touré a eu une histoire compliquée en tant que premier président de la Guinée, mais il s'est aussi illustré en rejetant les conditions néocoloniales de l'indépendance imposées par Charles de Gaulle en 1958.^43,44^ Aminata Sow Fall et Mariama Bâ, deux des premières romancières francophones africaines publiées, ont offert une critique sociale acerbe sur le statut des femmes sénégalaises et la propension de la société à rejeter les valeurs autochtones au profit de la culture française.^45,46^ Enfin, des commentaires récents de la domaine de la SSR émanant de professionnelles francophones ont démontré que la réalisation de l'égalité des sexes est inextricablement liée à une plus grande inclusion des francophones.^47,48^ Alors que nous continuons à débattre sur l'opérationnalisation du développement piloté par les acteurs locaux, tout en centrant notre travail sur les besoins exprimés par les femmes, il est problématique d'ignorer ces œuvres fondamentales simplement parce qu'elles sont publiées en français. Ce faisant, nous manquons des opportunités d'innovation et de progrès collectif.

Vers une conversation multilingue

En passant aux recommandations, nous réitérons notre plaidoyer en faveur de l'inclusion de la langue française comme première étape vers une inclusion linguistique plus large. Ne disposant pas de toutes les réponses, nous présentons ces recommandations afin de susciter un débat plus approfondi.

Que faire dès maintenant ?

Comme point de départ pour des initiatives plus larges d'inclusion linguistique de notre domaine, nous proposons les 4 actions clés suivantes que les organisations et les individu.e.s peuvent entreprendre dès maintenant pour soutenir une plus grande inclusion des professionnel(le)s francophones.

1. Utiliser des services de traduction assistée pour les communications électroniques quotidiennes avec les collaborateurs non anglophones.

De nombreuses applications fournissent des traductions suffisamment précises pour permettre les communications électroniques quotidiennes entre différentes langues. Certaines applications, comme DeepL, s'intègrent dans des logiciels existants et utilisent l'apprentissage automatique pour améliorer les traductions au fil du temps. Si vous ne parlez pas la langue de votre collaborateur, joignez le texte original à la traduction dans votre communication. Bien que la traduction assistée puisse être une alternative fonctionnelle pour les communications quotidiennes, nous recommandons toujours de faire appel à des traducteurs professionnels pour les traductions officielles et les publications externes.

2. Investir dans des services d'interprétation bidirectionnelle par des interprètes.

Les services d'interprétation sont devenus plus abordables, en particulier avec la prolifération des plateformes de réunions virtuelles. Bien que les services d'interprétation utilisant l'intelligence artificielle (IA) se soient considérablement améliorés, ils ne peuvent pas interpréter avec précision les nuances et les sensibilités de la langue dans le domaine de la SSR, en particulier entre les cultures ; par conséquent, nous ne recommandons pas actuellement l'interprétation par IA. Les organisations doivent prévoir un budget pour l'interprétation bidirectionnelle afin que chacun.e puisse s'exprimer dans la langue de son choix. Tester la qualité de l'interprétation et du son avec des sociétés d'interprétation avant un événement et conserver les mêmes interprètes pour plusieurs missions afin qu'ils puissent apprendre le langage propre à la discipline et à l'organisation. Cette approche est particulièrement importante dans le domaine de la SSR, qui comporte de nombreux termes sensibles et très contextualisés.

3. S'engager à intégrer la traduction de documents dans les processus habituels de gestion de projet.

Comme nous l'avons vu précédemment, l'absence de priorité systématique accordée à la traduction des documents entraîne des lacunes en matière d'information pour les populations francophones. Même les ressources fondamentales de la SSR ne sont souvent pas disponibles en français, ou leur traduction est de mauvaise qualité. Les professionnels SSR peuvent remédier à cette situation en intégrant les ressources clés et la traduction des produits finaux dans les calendriers de mise en œuvre des projets. Les anglophones ont également beaucoup à apprendre des travaux mis en œuvre dans les contextes francophones, raison pour laquelle la planification de la traduction du français vers l'anglais est également essentielle.

4. Concevoir des événements comme des espaces multilingues.

Fournir des services d'interprétation et de traduction est un premier pas vers une plus grande inclusion. Pour aller au-delà de l'inclusion et tendre vers l'appartenance, les organisations doivent concevoir des événements et des plateformes qui reconnaissent la diversité linguistique et démontrent aux non-anglophones qu'ils sont les bienvenus. Nous pouvons y parvenir de diverses manières, notamment par les moyens suivants.

- Traduire les diapositives et tout autre matériel de l'événement et les communiquer à l'avance aux interprètes et aux participants.
- Établir et maintenir des normes selon lesquelles tout le monde devrait utiliser les services d'interprétation, et pas seulement les non-anglophones. Dans un environnement dominé par l'anglais, les anglophones ne s'attendent souvent pas à des interventions en français et risquent de ne pas entendre ce que les francophones ont à dire s'ils n'écoutent pas l'interprétation.
- Faciliter les événements dans plusieurs langues, en alternant la langue que vous utilisez en premier ou le plus fréquemment.
- Inviter des présentateurs et des panélistes parlant différentes langues et les encourager à présenter leur exposé dans la langue dans laquelle ils sont le plus à l'aise.
- Encourager activement les échanges entre les langues par le biais de petits groupes mixtes et de l'apprentissage entre pairs.

Ce dont nous avons besoin pour un changement durable

Outre ces solutions plus immédiatement accessibles, nous proposons trois recommandations aux donateurs et aux organisations internationales pour promouvoir un changement structurel plus large, dont certaines s'appuient sur des recommandations déjà publiées.^2,26,29^

1. Promouvoir des systèmes de pouvoir inclusifs par le biais de 4 mécanismes principaux.

**Écouter la voix des francophones :** Cette démarche peut prendre plusieurs formes, mais la plus simple consiste à organiser des sessions d'écoute et des enquêtes afin d'entendre directement les francophones sur la manière dont ils souhaitent être impliqués et sur le rôle de l'inclusion linguistique dans le cadre de ces efforts. Investir dans de meilleurs services d'interprétation et de traduction pourrait être une solution, tout comme financer des cours d'anglais pour les francophones qui souhaitent apprendre cette langue. Les donateurs et les organisations internationales devraient réserver des fonds à ces solutions afin de les mettre en œuvre dès le départ.

**Rendre les possibilités de financement plus accessibles aux non-anglophones et imposer des services linguistiques de haute qualité tout au long du cycle de vie du projet :** Cela signifie qu'il faut publier les appels d'offres en anglais et dans la ou les langues principales du pays et permettre aux organisations de soumettre des propositions dans des langues autres que l'anglais. Cela signifie également que les considérations linguistiques doivent faire partie des conditions d'octroi des subventions, y compris l'affectation de fonds à la conception de programmes bilingues/multilingues, au soutien linguistique continu pendant la mise en œuvre et à la traduction de la documentation du projet.

**Prioriser le recrutement de personnel francophone à tous les postes :** Les changements culturels au sein de ces organisations peuvent entraîner des changements plus importants dans le domaine plus élargi. Donner la priorité au recrutement de personnes parlant couramment le français ou, idéalement, recruter davantage de francophones issus des pays bénéficiaires, nous permettrait d'atteindre un meilleur équilibre des pouvoirs. L'emploi direct de personnes originaires d'Afrique francophone peut également permettre de résoudre indirectement d'autres problèmes d'équité, car les groupes sous-représentés dans la sous-région ont moins de chances d'avoir la possibilité d'apprendre l'anglais que leurs homologues. En constituant un personnel multilingue, nous constituons également un personnel qui représente mieux les communautés au sein desquelles nous travaillons.

**Veiller à ce que les principaux organes de convocation et conférences dans le domaine de la SSR soient véritablement bilingues** : l'interprétation simultanée lors de ces événements est nécessaire mais insuffisante. Les francophones et ceux qui n'utilisent pas l'anglais comme langue de travail devraient être consultés et impliqués dans l'élaboration des plans de communication et d'événements. Les sessions doivent être proposées en anglais et en français, avec des présentations dans les deux langues et une interprétation bidirectionnelle pour les sessions de questions-réponses.

2. Investir dans des systèmes qui encouragent et évaluent en permanence la qualité des services de traduction et d'interprétation.

On part souvent du principe qu'il suffit d'offrir des services de traduction et d'interprétation, ce qui entraîne une certaine confusion lorsque les francophones ne participent pas de manière significative après la prestation de services. Comme nous l'avons vu précédemment, les systèmes de connaissances anglo-américains dominent le domaine de la SSR, propageant des concepts culturels typiquement américains tels que « autonomy » ou « empowerment », qui ne sont pas facilement traduits ou conceptualisés en français. Ainsi, nous soutenons que l'investissement dans les services linguistiques nécessite un investissement continu et permanent pour s'assurer que les interprètes et les traducteurs fournissent des traductions de qualité et nuancées d'idées complexes et évolutives. Pour bien faire, les organisations doivent investir dans des traducteurs et des interprètes internes ou régulièrement engagés, les préparer suffisamment pour les événements et gérer un lexique de termes et d'acronymes liés à la SSR et aux droits et spécifiques à l'organisation, avec leur traduction standard.

3. Institutionnaliser l'évolution vers une culture organisationnelle multilingue en intégrant les questions linguistiques dans les initiatives en matière de diversité, d'équité, d'inclusion et d'appartenance.

Pour se défaire plus de 50 ans d'hégémonie de l'anglais, nous avons besoin d'un investissement soutenu pour construire une culture où les non-anglophones se sentent à l'aise pour parler leur langue et où les anglophones n'adoptent pas par défaut l'anglais comme lingua franca. Vu l'importance accrue accordée à la diversité, à l'équité, à l'inclusion et à l'appartenance par les donateurs et les ONGI, nous pensons que les programmes de diversité, d'équité, d'inclusion et d'appartenance contiennent un potentiel inexploité pour promouvoir une évolution vers la valorisation du multilinguisme plutôt que de le considérer comme un obstacle à surmonter.

CONCLUSION

Bien que l'utilisation de l'anglais comme lingua franca dans le domaine de la SSR puisse faciliter les échanges entre pays, ce commentaire explique en quoi l'hégémonie de l'anglais contribue également à exclure une proportion importante de professionnels SSR qui utilisent le français comme langue de travail. Cette exclusion systémique alourdit le fardeau des groupes déjà défavorisés et finit par aller à l'encontre du mouvement de localisation auquel tant d'entre nous sont dévoués.

Pour combattre l'hégémonie de l'anglais, nous proposons des mesures simples que les individu.e.s peuvent entreprendre immédiatement, telles que la priorisation et la planification des services de traduction et d'interprétation et la conception d'espaces multilingues pour favoriser un sentiment d'appartenance. Nous préconisons également des changements structurels à long terme, réalisables en donnant la priorité à la représentation francophone dans les postes de pouvoir et en veillant à ce que les réunions mondiales soient réellement bilingues. Dans nos efforts continus pour améliorer les résultats SSR tout en luttant contre les inégalités au sein des professionnel(e)s de la SSR, nous devrions veiller à ce que l'inclusion linguistique fasse également partie de la conversation.

**Remerciements** : Nous remercions vivement les nombreux professionnels francophones de la SSR que nous avons consultés pour rédiger ce commentaire. Vos contributions ont fortement amélioré le commentaire et ont fait avancer notre réflexion sur une question complexe. Nous sommes reconnaissant.e.s aux personnes qui ont accepté de partager leurs expériences sous forme d'anecdotes au début de ce commentaire, ce qui a contribué à motiver et à façonner l'ensemble de l'article. Merci beaucoup.

**Contributions des auteurs** : ST : conceptualisation, supervision, rédaction de la version originale, rédaction, révision et édition. MF, MB, SAD, ML, EAP : révision et édition. EL : conceptualisation, validation, rédaction de la version originale, révision de la rédaction et édition. SN : conceptualisation, révision de la rédaction et édition . Tous les auteurs ont revu et approuvé la version finale du manuscrit en anglais et en français.

**Conflit d'intérêts** : Aucun n'a été déclaré.

Références

1. Tsuda Y. English hegemony and English divide. *China Media Res*. 2008;4(1):47-55.

2. Amano T. Les scientifiques dont l'anglais n'est pas la langue maternelle travaillent beaucoup plus dur pour suivre le rythme, selon une étude mondiale. The Conversation. 18 juillet 2023. Consulté le 23 septembre 2024. [https://theconversation.](https://theconversation.com/non-native-english-speaking-scientists-work-much-harder-just-to-keep-up-global-research-reveals-208750)com/non-native-english-speaking-scientists-work-much-harder-just-to-keep-up-global-research-reveals-208750

3. FP2030. Commitment Makers. Consulté le 23 septembre 2024, https://www.fp2030.org/commitment-makers/

4. Le Partenariat de Ouagadougou. Notre histoire. Consulté le 23 septembre 2024, https://partenariatouaga.org/notre-histoire/

5. Pays bénéficiaires de l'USAID pour la PF/SR. Agence américaine pour le développement international. Consulté le 23 septembre 2024, https://www.usaid.gov/global-health/health-areas/family-planning/countries

6. Planification familiale. Fondation Bill & Melinda Gates. Consulté le 23 septembre 2024. [https://www.](https://www.gatesfoundation.org/our-work/programs/gender-equality/family-planning)gatesfoundation.org/our-work/programs/gender-equality/family-planning

7. Agence américaine pour le développement international (USAID). *Aperçu du programme de planification familiale et de santé génésique*. USAID ; 2024. Consulté le 23 septembre 2024. [https://www.](https://www.usaid.gov/global-health/health-areas/family-planning/resources/family-planning-overview)usaid.gov/global-health/health-areas/family-planning/resources/family-planning-overview

8. Lemberg D. "The universal language of the future" : decolonization, development, and the American embrace of global English, 1945-1965. *Mod Intellect Hist*. 2018;15(2):561-592. [CrossRef](https://doi.org/10.1017/S1479244317000166)

9. Lemberg D. The end of empires and some linguistic turns : Les politiques linguistiques britanniques et françaises dans l'Afrique de l'entre-deux-guerres et de l'après-guerre. In : Fichter JR, ed. *British and French Colonialism in Africa, Asia and the Middle East : Connected Empires Across the Eighteenth to the Twentieth Centuries*. Springer International Publishing ; 2019:297-321.

10 Kasuya K. Discourses of linguistic dominance : a historical consideration of French language ideology. *Int Rev Educ*. 2001;47(3):235-251. [CrossRef](https://doi.org/10.1023/A:1017993507936)

11. Alidou H. Politiques linguistiques et éducation aux langues en Afrique francophone : Une critique et un appel à l'action. Dans : Makoni S, Smitherman G, Ball AF, Spears AK, eds : Makoni S, Smitherman G, Ball AF, Spears AK, eds. *Black Linguistics*. Routledge ; 2005:115-128.

12. Kamadjeu R. English : the lingua franca of scientific research. *Lancet Glob Health.* 2019;7(9):e1174. [CrossRef](https://doi.org/10.1016/S2214-109X(19)30258-X). [Medline](https://pubmed.ncbi.nlm.nih.gov/31401999)

13. Wexler A, Kates J, Lief E. *Donor Government Funding for Family Planning in 2021.* Kaiser Family Foundation ; 2022. Consulté le 23 septembre 2024. [https://www.](https://www.kff.org/report-section/donor-government-funding-for-family-planning-in-2021-report/)kff.org/report-section/donor-government-funding-for-family-planning-in-2021-report/

14. Finances. PC 2030. Consulté le 23 septembre 2024. [https://progress.](https://progress.fp2030.org/finance/)fp2030.org/finance/

15. RDC. FP Country Action Process EvaluationAccessed September 23, 2024. [https://www.](https://www.fpcape.org/countries/drc/)fpcape.org/countries/drc/

16. Pays et langues. Nations en ligne. Consulté le 23 septembre 2024. [https://www.](https://www.nationsonline.org/oneworld/countries_by_languages.htm)nationsonline.org/oneworld/countries_by_languages.htm

17. Introduction aux langues africaines. Université de Harvard. Consulté le 23 septembre 2024. [https://alp.](https://alp.fas.harvard.edu/introduction-african-languages)fas.harvard.edu/introduction-african-languages

18. Quels sont les continents où l'on trouve le plus de langues indigènes ? Ethnologue. Consulté le 23 septembre 2024. [https://www.](https://www.ethnologue.com/insights/continents-most-indigenous-languages/)ethnologue.com/insights/continents-most-indigenous-languages/

19. Ida IO. *L'alphabétisation au Niger : une analyse à partir des données du recensement de 2012*. Observatoire démographique et statistique de l'espace francophone ; avril 2015. Consulté le 23 septembre 2024. [https://numerique.](https://numerique.banq.qc.ca/patrimoine/details/52327/3799232)banq.qc.ca/patrimoine/details/52327/3799232

20 Lee A, Schultz KA. Comparing British and French colonial legacies : a discontinuity analysis of Cameroon. *Q J Politic Sci*. 2012;7(4):365-410. CrossRef

21 Nwude EC, Ugwuegbe SU, Adegbayibi AT. Impact du niveau de revenu et de l'aide étrangère sur la croissance économique en Afrique subsaharienne : le cas des pays anglophones et francophones. *Economic Research-Ekonomska Istraživanja*. 2023;36(2). [CrossRef](https://doi.org/10.1080/1331677X.2022.2142826)

22. Gouëset C. La Françafrique de Nicolas Sarkozy, changement... et continuité. *L'Express*. Consulté le 23 septembre 2024. [https://www.](https://www.lexpress.fr/monde/afrique/la-francafrique-de-nicolas-sarkozy-changement-et-continuite_851223.html)lexpress.fr/monde/afrique/la-francafrique-de-nicolas-sarkozy-changement-et-continuite_851223.html

23. Sagno G. Françafrique : la politique africaine de la France de Mitterrand à Macron. *BBC News Afrique*. Consulté le 23 septembre 2024. [https://www.](https://www.bbc.com/afrique/region-42144947)bbc.com/afrique/region-42144947

24. El Bcheraoui C, Mimche H, Miangotar Y, et al. Burden of disease in francophone Africa, 1990-2017 : a systematic analysis for the Global Burden of Disease Study 2017. *Lancet Glob Health*. 2020;8(3):e341-e351. [CrossRef](https://doi.org/10.1016/S2214-109X(20)30024-3). [Medline](https://pubmed.ncbi.nlm.nih.gov/32087171)

25 Liang M, Simelane S, Fortuny Fillo G, et al. The state of adolescent sexual and reproductive health. *J Adol Health*. 2019;65(6 Suppl):S3-S15. [CrossRef](https://doi.org/10.1016/j.jadohealth.2019.09.015). [Medline](https://pubmed.ncbi.nlm.nih.gov/31761002)

26. Arenas-Castro H. Des revues prestigieuses compliquent la tâche des scientifiques qui ne parlent pas anglais. Et nous sommes tous perdants. The Conversation. 21 mars 2024. Consulté le 23 septembre 2024. [https://theconversation.](https://theconversation.com/prestigious-journals-make-it-hard-for-scientists-who-dont-speak-english-to-get-published-and-we-all-lose-out-226225)com/prestigious-journals-make-it-hard-for-scientists-who-dont-speak-english-to-get-published-and-we-all-lose-out-226225

27. Roca A, Boum Y, Wachsmuth I. Plaidoyer contre l'exclusion des francophones dans la recherche en santé mondiale. [Article en français]. *Lancet Glob Health*. 2019;7(6):e701-e702. [CrossRef](https://doi.org/10.1016/S2214-109X(19)30175-5). [Medline](https://pubmed.ncbi.nlm.nih.gov/31000374)

28. Büyüm AM, Kenney C, Koris A, Mkumba L, Raveendran Y. Décoloniser la santé mondiale : si ce n'est pas maintenant, quand ? *BMJ Glob Health*. 2020;5(8):e003394. [CrossRef](https://doi.org/10.1136/bmjgh-2020-003394). [Medline](https://pubmed.ncbi.nlm.nih.gov/32759186)

29. Affun-Adegbulu C, Adegbulu O. Decolonising global (public) health : from Western universalism to global pluriversalities. *BMJ Glob Health*. 2020;5(8):e002947. [CrossRef](https://doi.org/10.1136/bmjgh-2020-002947). [Medline](https://pubmed.ncbi.nlm.nih.gov/32819916)

30. Abimbola S, Asthana S, Montenegro C, et al. Addressing power asymmetries in global health : imperatives in the wake of the COVID-19 pandemic. *PLoS Med.* 2021;18(4):e1003604. [CrossRef](https://doi.org/10.1371/journal.pmed.1003604). [Medline](https://pubmed.ncbi.nlm.nih.gov/33886540)

31. Nkrumah K. *Néo-colonialisme*. Nelson ; 1965.

32. Agence américaine pour le développement international (USAID). *La localisation à l'USAID : The Vision and Approach*. USAID ; 2022. Consulté le 23 septembre 2024. [https://www.](https://www.usaid.gov/sites/default/files/2022-12/USAIDs_Localization_Vision-508.pdf)usaid.gov/sites/default/files/2022-12/USAIDs_Localization_Vision-508.pdf

33. Diversité, équité et inclusion. Fondation Bill & Melinda Gates. Consulté le 23 septembre 2024. [https://www.](https://www.gatesfoundation.org/about/diversity-equity-inclusion)gatesfoundation.org/about/diversity-equity-inclusion

34. Wishart W. Words matter : that's why Oxfam is launching an inclusive language guide. 13 mars 2023. Consulté le 23 septembre 2024. [https://views-voices.](https://views-voices.oxfam.org.uk/2023/03/launch-inclusive-language-guide/)oxfam.org.uk/2023/03/launch-inclusive-language-guide/

35. Piller I, Zhang J, Li J. La diversité linguistique en temps de crise : les défis linguistiques de la pandémie COVID-19. *Multilingua*. 2020;39(5):503-515. [CrossRef](https://doi.org/10.1515/multi-2020-0136)

36. Mogaka OF, Stewart J, Bukusi E. Why and for whom are we decolonising global health ? *Lancet Glob Health*. 2021;9(10):e1359-e1360. [CrossRef](https://doi.org/10.1016/S2214-109X(21)00317-X). [Medline](https://pubmed.ncbi.nlm.nih.gov/34534477)

37. Pennycook A. Le mythe de l'anglais comme langue internationale. In : Sinfree M, Alastair P, eds. *Disinventing and Reconstituting Languages*. Multilingual Matters ; 2006:90-115.

38 Pérez-Llantada C. Bringing into focus multilingual realities : faculty perceptions of academic languages on campus. *Lingua*. 2018;212:30-43. [CrossRef](https://doi.org/10.1016/j.lingua.2018.05.006)

39. The Lancet Public Health. Deux jours à Abidjan : trouver la voix de l'Afrique francophone. *Lancet Public Health*. 2017;2(2):e56. [CrossRef](https://doi.org/10.1016/S2468-2667(17)30015-4). [Medline](https://pubmed.ncbi.nlm.nih.gov/29253390)

40. Phillipson R. *Linguistic Imperialism Continued*. Routledge ; 2013.

41 Cleaver S, Grimard NO, Balit J, Gautier L, Lubin-Jerome L. Language hegemony in decolonising global health conferences. Consulté le 23 septembre 2024. [https://escholarship.](https://escholarship.mcgill.ca/concern/articles/1v53k268p)mcgill.ca/concern/articles/1v53k268p

42 Harsch E. The legacies of Thomas Sankara : a revolutionary experience in retrospect. *Revr Afr Politic Econ*. 2013;40(137):358-374. [CrossRef](https://doi.org/10.1080/03056244.2013.816947)

43. Magnan P. 28 septembre 1958 : le jour où la Guinée a dit non à de Gaulle. *franceinfo*. 10 mai 2018. Consulté le 23 septembre 2024. [https://www.](https://www.francetvinfo.fr/monde/afrique/politique-africaine/28-septembre-1958-le-jour-ou-la-guinee-a-dit-non-a-de-gaulle_3055865.html)francetvinfo.fr/monde/afrique/politique-africaine/28-septembre-1958-le-jour-ou-la-guinee-a-dit-non-a-de-gaulle_3055865.html

44. Eboulé C. Guinée : la souveraineté monétaire, une histoire mouvementée. 9 mars 2020. Consulté le 23 septembre 2024. [https://information.](https://information.tv5monde.com/afrique/guinee-la-souverainete-monetaire-une-histoire-mouvementee-32676)tv5monde.com/afrique/guinee-la-souverainete-monetaire-une-histoire-mouvementee-32676

45. Ajala JD. La grève des mendiants" : Aminata Sow Fall, porte-parole des déshérités. *CLA J.* 1990;34(2):137-152.

46. Latha RH. Les féminismes dans un contexte africain : La si longue lettre de Mariama Bâ. *Agenda : Empower Women Gender Equity.* 2001 ;(50):23-40.

47. Ouédraogo S, Gautier L, Mac-Seing M, et al. De-patriarchalising and levelling science for French-speaking women. *Lancet*. 2019;393(10171):e23-e24. [CrossRef](https://doi.org/10.1016/S0140-6736(18)32092-0). [Medline](https://pubmed.ncbi.nlm.nih.gov/30739705)

48. Jones CM, Gautier L, Kadio K, et al. Equity in the gender equality movement in global health. *Lancet*. 2018;392(10142):e2-e3. [CrossRef](https://doi.org/10.1016/S0140-6736(18)31561-7). [Medline](https://pubmed.ncbi.nlm.nih.gov/30017139)

Révision par les pairs

**Reçu** : 10 janvier 2024 ; **Accepté** : 18 septembre 2024 ; **Première publication en ligne** :

**Citez cet article comme suit** : Turke S, Fall M, Ba M, et al. "Je suis désolé, je parle français" : comment l'hégémonie de l'anglais sape les efforts pour changer le pouvoir dans la santé mondiale. *Glob Health Sci Pract*. 2024;12(5):e2400201. <https://doi.org/10.9745/GHSP-D-24-00201>

© Turke et al. Ceci est un article en libre accès distribué selon les termes de la licence Creative Commons Attribution 4.0 International License (CC BY 4.0), qui permet l'utilisation, la distribution et la reproduction sans restriction sur n'importe quel support, à condition que l'auteur original et la source soient correctement cités. Pour consulter une copie de la licence, visitez le site https://creativecommons.org/licenses/by/4.0/. Lorsque vous créez un lien vers cet article, veuillez utiliser le lien permanent suivant [: https://doi.org/10.9745/GHSP-D-24-00201](https://doi.org/10.9745/GHSP-D-24-00201)

1. Jhpiego, Baltimore, MD, États-Unis.

   ^b^ Consultante indépendante, Dakar, Sénégal.

   ^c^ Unité de Coordination du Partenariat de Ouagadougou, Dakar, Sénégal.

   ^d^ Engineering & Environment Services, Dakar, Senegal.

   ^e^ EngenderHealth Afrique de l'Ouest et du Centre, Abidjan, Côte d'Ivoire.

   ^f^ Center on Gender Equity and Health, University of California San Diego, San Diego, CA, États-Unis.

   ^g^ Garabam Consulting, Livingston, TX, États-Unis.

   Correspondance avec Shani Turke ([shani.turke07@gmail.com](mailto:shani.turke@jhpiego.org)). [↑](#footnote-ref-1)
